# The effect of environmental heterogeneity on species richness depends on community position along the environmental gradient

**DOI:** 10.1038/srep15723

**Published:** 2015-10-28

**Authors:** Zhiyong Yang, Xueqi Liu, Mohua Zhou, Dexiecuo Ai, Gang Wang, Youshi Wang, Chengjin Chu, Jeremy T. Lundholm

**Affiliations:** 1 State Key Laboratory of Grassland and Agro-Ecosystems, School of Life Sciences, Lanzhou University, Lanzhou, 730000, China; 2 Ministry of Education Key Laboratory of Western China’s Environmental Systems, Research School of Arid Environment and Climate Change, Lanzhou University, Lanzhou, 730000, China; 3 SYSU-Alberta Joint Lab for Biodiversity Conservation, State Key Laboratory of Biocontrol and School of Life Sciences, Sun Yat-sen University, Guangzhou, 510275, China; 4 Department of Biology/Environmental Studies Program, Saint Mary’s University, Halifax, Nova Scotia, Canada B3H3C3

## Abstract

Environmental heterogeneity is among the most important factors governing community structure. Besides the widespread evidence supporting positive relationships between richness and environmental heterogeneity, negative and unimodal relationships have also been reported. However, few studies have attempted to test the role of the heterogeneity on species richness after removing the confounding effect of resource availability or environmental severity. Here we constructed an individual-based spatially explicit model incorporating a long-recognized tradeoff between competitive ability and stress-tolerance ability of species. We explored the impact of the level of resource availability (i.e. the position of the community along a gradient of environmental severity) on the heterogeneity-diversity relationship (HDR). The results indicate that the shape of HDR depends on the community position along the environmental gradient: at either end of the gradient of environmental severity, a positive HDR occurred, whereas at the intermediate levels of the gradient, a unimodal HDR emerged. Our exploration demonstrates that resource availability/environmental severity should be considered as a potential factor influencing the shape of the HDR. Our theoretical predictions represent hypotheses in need of further empirical study.

Environmental heterogeneity is ubiquitous in natural systems and influences population dynamics and community structure^1^,^2^,^3^,^4^,^5^,^6^,^7^. Among ecological hypotheses relevant to environmental heterogeneity, the shape of the heterogeneity-diversity relationship (HDR) has been intensively explored in the past decades^2^,^8^,^9^,^10^,^11^,^12^,^13^. Based on the niche differentiation concept, a more heterogeneous environment could support more species through partitioned niche space^1^,^2^,^3^,^14^,^15^,^16^,^17^,^18^,^19^, which implies a positive HDR. Though positive HDRs have been widely documented in literature, several studies have recently questioned the generality of this pattern^20^,^21^,^22^,^23^,^24^. Other types of HDR, including negative, unimodal and non-significant, have been frequently reported as well^24^,^25^,^26^,^27^,^28^.

In plant communities, mean resource levels can affect species richness at fine spatial scales^9^. Many experimental studies have separated the effects of resource heterogeneity from average resource availabilities^25^, but the influence of spatial environmental heterogeneity across longer gradients of habitat fertility has been little examined. A previous study showed that intra-plot environmental heterogeneity should allow for more species to coexist in plots than predicted by mean levels of environmental variables^29^. Multiple factors, such as different measures of heterogeneity and spatial scale^12^,^13^,^25^,^26^, have been invoked to explain variation in the shape of HDRs. However, the effect of average resource availability on HDRs has been completely overlooked in previous studies of environmental gradients^26^.

Many studies have shown that environmental severity can exert a strong influence on species richness^1^,^30^,^31^. In temperate and boreal biomes, the relationship between plant species richness and biomass tends to be unimodal, whereas in the tropics it is often monotonically increasing^32^. Mechanisms determining these patterns are still debated^33^,^34^,^35^. To explain unimodal richness-biomass patterns, Grime invoked local plant interactions (competition) as reducing diversity in situations with low environmental severity, whereas richness declines in areas with high environmental severity due to lower numbers of species in regional pools that can tolerate stressful conditions^30^. Others have suggested that smaller regional species pools for the extremes of environmental gradients combined with larger pools for intermediate environmental conditions can cause unimodal diversity patterns^36^,^37^. Varying dispersal limitations for species associated with different parts of environmental gradients may also contribute to richness patterns^38^.

The relationships between environmental heterogeneity and species diversity across environmental gradients should depend on the mechanisms governing the overall relationship between mean environmental severity and species richness and the effect of heterogeneity on diversity at a given level of mean environmental severity.

More heterogeneous patches will contain a greater range of conditions at the same mean level of environmental severity. The increasing range of environmental conditions, however, does not necessarily result in higher species richness. In fact, the impact of the increasing range of environmental conditions on biodiversity will largely depend on the relationship between environmental severity and richness, as the number of species each heterogeneous patch can support corresponds to a specific location with the same level of environmental severity of this heterogeneous patch on the environmental severity-richness relationship obtained from homogeneous landscapes. This implies that the shape of the HDR could vary along environmental gradients. However, no studies have explored this hypothesis, theoretically or experimentally.

In the present paper, through an individual-based spatially explicit model based on previous studies^39^,^40^, we control the level of resource availability, and isolate the effect of environmental heterogeneity from that of resource availability on community structure. This model simulates communities of sessile organisms and generates unimodal species richness-environmental severity relationships, based on the trade-off between stress-tolerance and competitive ability^30^. In the model, environmental severity is negatively associated with resource availability (represented by a variable *S* in the model)^39^, i.e. environmental severity is maximal at the lowest resource availability or productivity. The shape of the species richness-environmental severity relationship in this model is fairly robust to variation in species pool size^40^. We hypothesized that the shape of HDR would depend on the location of communities along environmental gradients: at either end of the environmental gradient, heterogeneity should promote greater richness, i.e. a positive HDR, as it will increase the prevalence of patches with intermediate environmental severity which draw from the largest species pool; at intermediate severity levels, heterogeneity could promote lower richness, i.e. a negative HDR, as patches will be present that draw from the smaller species pools associated with environmental extremes.

## Results

**Figure 1:**
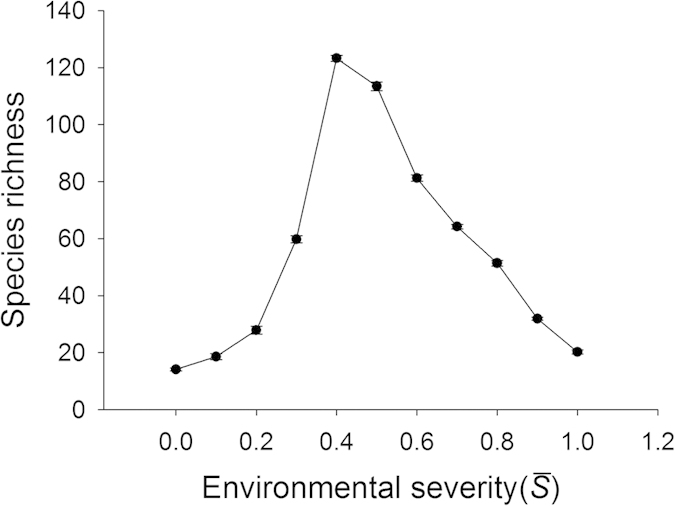
Change of species richness along the environmental severity gradient. Landscapes were homogeneous with the size of 100 × 100 cells. Each data point represents the mean ± SE (N = 10). The parameter values used in models are *r*_*max*_ = 1, *r*_*min*_ = 0.2, *r*_*s*_ = 0.1 and *c* = 1.

On the homogenous landscapes without heterogeneity, as displayed by other studies using this model^39^,^40^, there existed a hump-shaped relationship between environmental severity and species richness, with the highest value occurring at intermediate environmental conditions (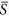 is about 0.4) (Fig. 1). However, the curve shapes within each half of this humped relationship were different. For the left half of this hump-shaped curve (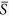 is from 0.0 to 0.4), the change of species richness (i.e. the slope of the curve at a given environmental severity) arose as an increasing function with environmental severity. For the right half of the curve (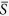 is from 0.4 to 1.0), however, the change of species richness was relatively constant with environmental severity.

**Figure 2:**
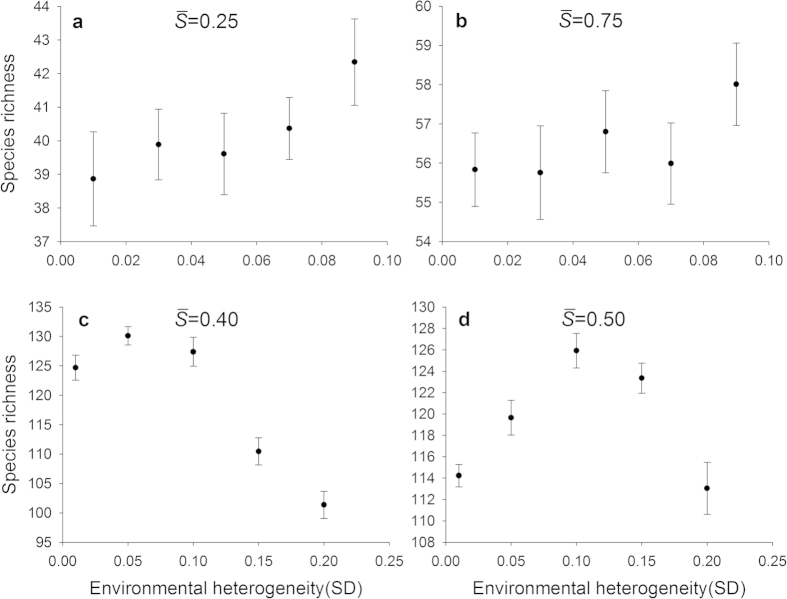
Comparison of species richness-environmental heterogeneity relationships. Environmental heterogeneity was represented by the standard deviation of *S*_*k*_ values across patches. The environmental severity (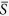) was 0.25 in (**a**), 0.75 in (**b**), 0.40 in (**c**), and 0.50 in (**d**). The whole landscape was divided into 400 patch types. Each data point represents the mean ± SE (N = 10).

All comparisons relevant to the heterogeneous landscapes were under the constraint of the equal average level of environmental severity. In the case of the average environmental severity equal to 0.25 and 0.75 (Fig. 2a,b), there was a positive trend between species richness and environmental heterogeneity (represented by the standard deviation of *S*_*k*_ across patches), though it was not substantial. In contrast, when the average environmental severity was equal to 0.40 and 0.50, species richness first increased then decreased with the standard deviation of *S*_*k*_ across patches, leading to a unimodal relationship between richness and environmental heterogeneity (Fig. 2c,d).

## Discussion

When mean environmental severity or resource availability was held constant, our results demonstrated that the shape of the HDR was influenced by the positions of communities along the resource availability gradient: a positive HDR when resource availability was near the ends of the environmental gradient, and a unimodal HDR at the intermediate levels of environmental severity.

These divergent patterns could be largely explained by the relationship between species richness and environmental severity on homogeneous landscapes (Fig. 1). For example, in the case where the average environmental severity was 0.25 (Fig. 2a), richness increased with increasing heterogeneity (SD). Along that range of environmental severity under homogeneous conditions (Fig. 1), richness increased strongly with environmental severity between 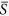 = 0.2 and 0.3, but less strongly between 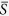 = 0.1 and 0.2. Thus under heterogeneous conditions at environmental severity 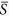 = 0.25 (Fig. 2a), the increase of species richness in patches with 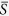 values approaching 0.50 was partly offset by the decrease of species richness in patches with 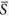 values approaching 0.0. Consequently, environmental heterogeneity led to an increase in species richness (Fig. 2a). In a similar way, for the case of 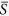 = 0.75, there also existed a positive trend in HDR (Fig. 2b). The results with the positive HDR are consistent with many previous studies^12^,^25^,^26^,^41^, and support our hypothesis that a positive HDR would occur at the ends of the environmental gradient.

Our hypothesis that a negative HDR would emerge at the intermediate levels of the environmental gradient was partly supported by the simulation results. Actually, a unimodal rather than monotonic HDR occurred (Fig. 2c,d). Take 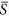 = 0.40 as an example. When the standard deviation was small, such as 0.01, the *S*_*k*_ values for 400 patches were closely clustered around the mean 0.4, covering a very small range of potential environmental niches. The hump-shaped curve of species richness versus environmental severity (Fig. 1) implies that for a given area, a patch characterized by an intermediate level of environmental severity would support more species than the one characterized by other levels of severity. With the increase of the standard deviation, the landscape spanned a wider environmental range, resulting in higher species richness. However, with the further increase of the standard deviation, each patch had an increasing probability to be assigned an *S*_*k*_ value near two ends of the gradient. Thus, the increasing proportion of patches on the landscape with *S*_*k*_ values near two ends of the gradient inevitably led to a decrease in species richness (Fig. 1).

Negative HDRs have been a key topic in recent studies^16^,^20^,^22^,^27^,^28^. If high heterogeneity also results in smaller habitats characterized by similar conditions, then overall richness can decline due to stochastic extinctions^42^. This is now called “microfragmentation” and is invoked to explain negative HDRs^20^,^22^,^26^,^27^,^28^. Within a fixed total community size, species richness decreases due to the stochastic extinction at high levels of environmental heterogeneity because the area available per habitat decreases^43^. However, the mechanism of microfragmentation did not operate in our simulations, as the number of patches (400 patches) and the area of landscape (100 × 100 cells) were both fixed. This implies that the present study provides a novel explanation for the negative HDR, which needs further empirical tests. Additionally, species richness is usually closely associated with the spatial extent of the study^44^,^45^. One potential refinement of our model could explore the combined effects of landscape area, resource availability and heterogeneity on community dynamics and their interactions.

Our results demonstrate that the shape of the relationship between species richness and environmental heterogeneity strongly depends on the positions of communities located on gradients of environmental severity. The experimental tests for predictions explored here could be easily implemented in field. A recent experimental study^27^ provides a good example showing how to isolate the confounding effect of resource availability from resource heterogeneity on species richness^27^. Additionally, because growth rate (and likely, the rates of competitive exclusion) and individual size (individual plants are larger in lower environmental severity) usually change with the environmental gradient^46^, a refined version of our model should explicitly consider plant size and the potential relationship between the spatial scale and the size of individual plants^25^,^26^,^47^. In the present work, we tested the change of HDR in the context of the unimodal environmental severity-diversity pattern. Another prospective improvement of the model is to explore the potential influence of various environmental severity-diversity relationships on HDRs, to determine the generality of the conclusions obtained here.

## Methods

Our model involves a long-recognized trade-off between competitive ability and stress tolerance of plant species^30^,^37^,^39^,^48^,^49^,^50^,^51^,^52^,^53^. In the model, species display a strategy-dependent distribution along the environmental gradient: Those with strong competitive ability would dominate communities in benign conditions (low environmental severity), while ones with strong stress-tolerant ability would dominate communities in harsh conditions (high environmental severity). This model supplies a unique position for us to test HDR, as it explicitly incorporates the linkage between the strategy of life history closely associated with species’ niche requirements and environmental gradients.

In our model, environmental severity was patch-specific (the variable *S*_*k*_ was designated for patch *k*), with the range from 0 (i.e. the most benign environment) to 1 (i.e. the most severe environment). A variable *p*_*j, i*_ represents the competitive ability of individual *j* of species *i*. To obtain *p*_*j, i*_ values, we firstly randomly drew a value for each species (*p*_*i*_) from a uniform distribution [0, 1] with the interval of 0.005 (1 divided by the size of regional species pool). This set of values for all species represents inter-specific variation. Then, for each individual of a given species, we randomly drew a value from a normal distribution, with the mean equal to *p*_*i*_ and the standard deviation of 0.003. Through this, we incorporated intra-specific variation into the model. The results from skewed distributions of competitive abilities were qualitatively similar with the ones presented here (see the Supplementary Figs S1–S4 online). The larger the *p*_*j, i*_ is, the higher the probability that species *i* invades a neighboring cell occupied by another species. We also assumed that any species could invade the empty cells. To account for the tradeoff, we assumed that the reproduction rate (*r*) of competitive species declines more rapidly with increasing environmental severity than that of stress-tolerant species:


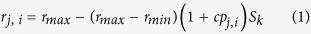


where *r*_*max*_ and *r*_*min*_ refer to the maximum and minimum reproductive rate, respectively, which were equal among species and *c* was a constant greater than 0, indicating the linear negative correlation between the competitive ability and the fecundity of species. Though it is possible to set species-specific *r*_*max*_ and *r*_*min*_, we have no sufficient prior information from literature to determine the relationships between *r*_*max*_ and *r*_*min*_ and corresponding competitive ability of species. When an individual’s reproductive rate *r* was negative but greater than a threshold *r*_*s*_, it could reproduce but still persisted in the community. If an individual’s reproductive rate *r* was more negative than *r*_*s*_, the individual died^39^,^54^.

Simulation modeling was executed on a two-dimensional square landscape with the size of 100 × 100 cells. Each cell can be empty or occupied by only one individual plant. Different measures for environmental heterogeneity have been proposed in literature^12^,^13^, including diversity of land cover types^16^, elevation range^22^, and the standard deviation of a specific environmental variable^55^. In the present paper, without loss of generality, we quantified environmental heterogeneity through the standard deviation of *S*_*k*_ across patches on the landscape^42^, thus heterogeneity and species diversity were evaluated for the entire landscape. Thus, the homogenous landscape means that the standard deviation of *S*_*k*_ across patches was equal to 0. We explored the change of species richness along the environmental gradient on these homogeneous landscapes.

In terms of heterogeneity, we divided the landscape into 400 patches, with each patch having 25 cells (the results from the case with 2500 patches were qualitatively similar with the ones presented here). To test whether HDR changes along the environmental gradient, we considered four levels of resource availability without loss of generality, i.e. 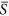 = 0.25, 0.40, 0.50, and 0.75. Via changing the standard deviation, we obtained the different levels of heterogeneity. Hence, for each patch, the variable of environmental severity, i.e. *S*_*k*_, was randomly drawn from a normal distribution with 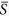 parameterized here and the standard deviation of interest. In other words, patches differed with respect to *S*_*k*_ values, but cells within a given patch shared the identical value. For the cases of 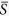 = 0.25 and 0.75, we set the standard deviation as 0.01, 0.03, 0.05, 0.07, and 0.09. For the case of 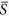 = 0.40 and *S* = 0.50, standard deviation was set as 0.01, 0.05, 0.10, 0.15 and 0.20. Such settings of standard deviation guarantee that most values randomly drawn from normal distributions fall into the range of the environmental gradient [0, 1].

The initial landscape was saturated and occupied by the same number of species as in regional species pool (*R* = 200 here; the size of regional species pool will not qualitatively change the results), with the number of individuals for each species following a negative exponential distribution. The model sequentially ran through the following modules: mortality, immigration, and reproduction and dispersal.

### Mortality

All individuals in the community suffered from a given degree of environmental stochasticity and disturbance. To remove the potential confounding effect of mortality from environmental heterogeneity, we assumed that individuals from all species experience the same probability of death due to stochastic factors.

### Immigration

In each iteration, we randomly selected a fixed number (*I* = 10; results are qualitatively similar with *I* = 20) of individuals from the regional species pool *R*. These selected individuals were randomly assigned to cells across the whole landscape. When the cell an individual reached was occupied by another individual, the relative competitive ability between species to which the two individuals belonged determined whether the invading individual could exclude the resident individual and occupy the target cell. For instance, for individual *m* of resident species *k* and individual *j* of invading species *i*, if *p*_*j,i*_ − *p*_*m,k*_ was greater than a random value drawn from a uniform distribution from 0 to 1, the individual of species *i* replaced the individual of species *j*.

### Reproduction and dispersal

Equation 1 determined the reproductive rate *r*_*j,i*_ for each individual. As *r*_*j,i*_ is not always an integer, we calculated the number of offspring with the following rule: If the fractional part of *r*_*j,i*_ is greater than a random value drawn from a uniform distribution ([0, 1]), the number of offspring is equal to the integer part of *r*_*j,i*_ plus 1. The competitive ability of offspring was identical to the parent. These newly born propagules were randomly dispersed to the neighboring eight cells of the parent. We assumed that the dispersal can occur within each patch, or among patches. Similar to the above rule of immigration, if the cell the propagule reached was occupied by an individual of another species, the competitive ability between them determined the final outcome. In addition, the propagules of all species could successfully invade the empty cells.

Simulations were run 10 000 steps in order to allow the community to approach a dynamical equilibrium state. Community composition, including species identity and abundance, was recorded in 200 step intervals for each setting of parameters after the 10 000 startup steps. We conducted 10 replicates for each setting of parameters. Simulations were implemented in NetLogo (v5.0.4) software^56^, and a “wrap-around” approach was used to avoid edge effects^57^.

## Additional Information

**How to cite this article**: Yang, Z. *et al*. The effect of environmental heterogeneity on species richness depends on community position along the environmental gradient. *Sci. Rep.* **5**, 15723; doi: 10.1038/srep15723 (2015).

## Supplementary Material

Supplementary Information
