# Supplementary material for: The effect of environmental heterogeneity on species richness depends on community position along the environmental gradient
Source: Sci Rep. 2015 Oct 28;5:15723. doi: 10.1038/srep15723 (PMC4623746; doi:10.1038/srep15723)
Supplement: Supplementary Information [file srep15723-s1.pdf]

Supplementary Information

The effect of environmental heterogeneity on species richness depends on community position along the environmental gradient

Zhiyong Yang[1], Xueqi Liu[1], Mohua Zhou[1], Dexiecuo Ai[1], Gang Wang[1], Youshi Wang[2,*], Chengjin Chu[1,3,*], Jeremy T. Lundholm[4]

[1] State Key Laboratory of Grassland and Agro-Ecosystems, School of Life Sciences, Lanzhou University, Lanzhou, 730000, China

[2] Ministry of Education Key Laboratory of Western China's Environmental Systems, Research School of Arid Environment and Climate Change, Lanzhou University, Lanzhou, 730000, China

[3] SYSU-Alberta Joint Lab for Biodiversity Conservation, State Key Laboratory of Biocontrol and School of Life Sciences, Sun Yat-sen University, Guangzhou, 510275, China

[4] Department of Biology/Environmental Studies Program, Saint Mary's University, Halifax, Nova Scotia, Canada B3H3C3

* Correspondence and requests for materials should be addressed to:

Y. Wang, email: wangysh@lzu.edu.cn; C. Chu, email: cjchu@lzu.edu.cn

We explored two extreme scenarios with the skewed distribution of competitive ability of species to explore sensitivity of the models to variation in the distribution of traits within the species pool. We varied the distribution of competitive abilities along the competitive ability-stress tolerance trade-off in two ways: (a) competitive abilities of 20% species were uniformly distributed between 0 and 0.5, and competitive abilities of 80% species were uniformly distributed between 0.5 and 1 (Figs. S1 and S2), and (b) competitive abilities of 80% species were uniformly distributed between 0 and 0.5, competitive abilities of 20% species were uniformly distributed between 0.5 and 1 (Figs. S3 and S4).

(a) 20% of species were uniformly distributed between 0 and 0.5, and 80% of species uniformly distributed between 0.5 and 1.

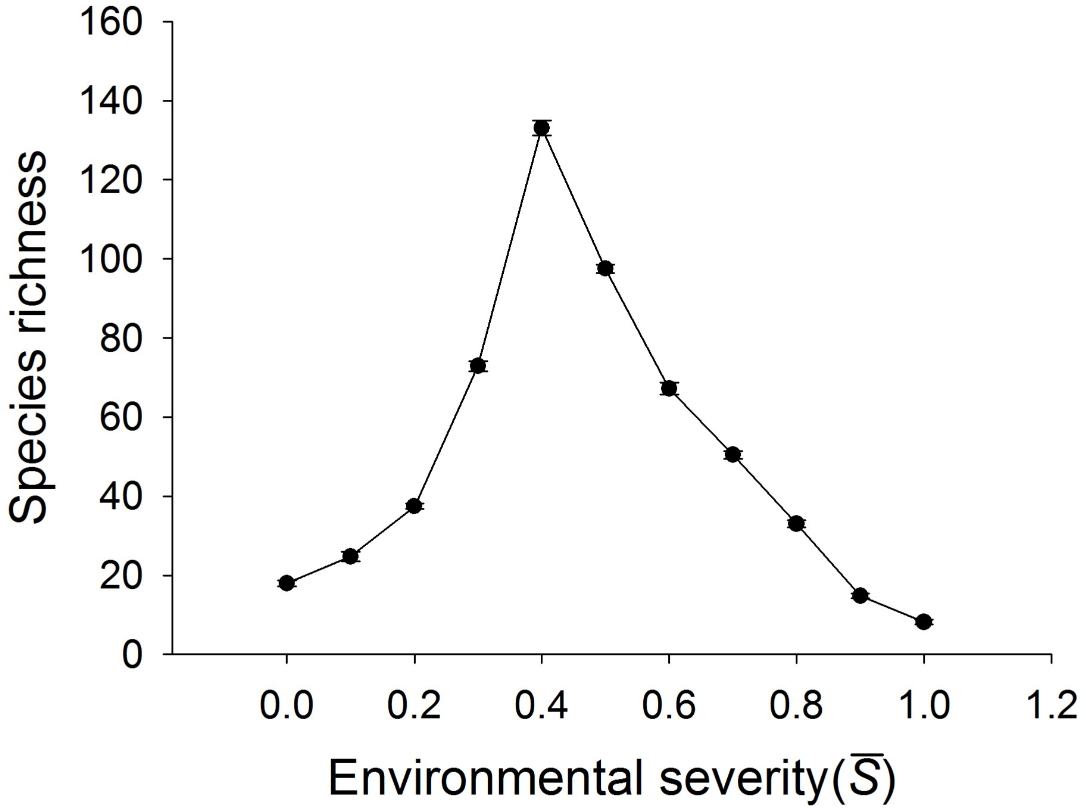

Supplementary Figure S1. Change of species richness along the environmental severity gradient. Landscapes were homogeneous with the size of 100 × 100 cells. Each data point in the panel represents the mean ± SE (N = 10). The parameter values used in models are r_{max} = 1, r_{min} = 0.2, r_s = 0.1 and c = 1.

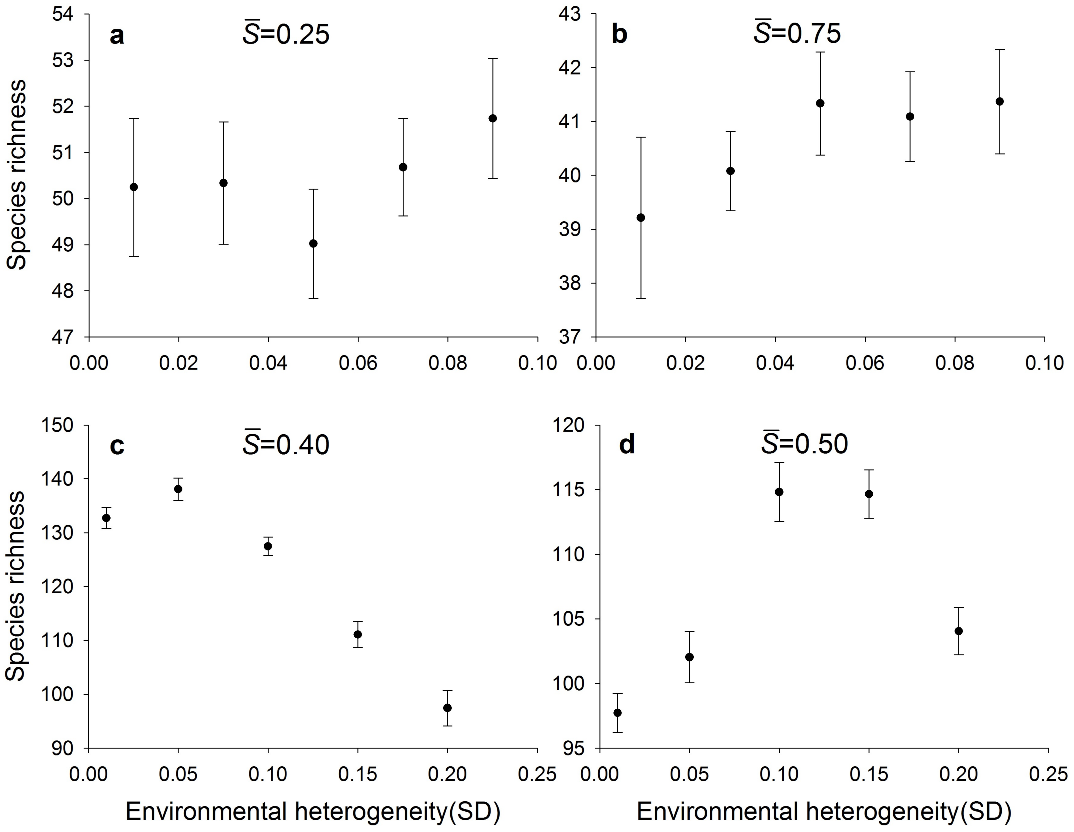

Supplementary Figure S2. Comparison of species richness-environmental heterogeneity relationships. Environmental heterogeneity was represented by the standard deviation of S_k values across patches. The environment severity ($\bar{S}$) was 0.25 in (**a**), 0.75 in (**b**), 0.40 in (**c**), and 0.50 in (**d**). The whole landscape was divided into 400 patch types. Each data point in panels represents the mean ± SE (N = 10).

(b) 80% of species were uniformly distributed between 0 and 0.5, and 20% of species uniformly distributed between 0.5 and 1.

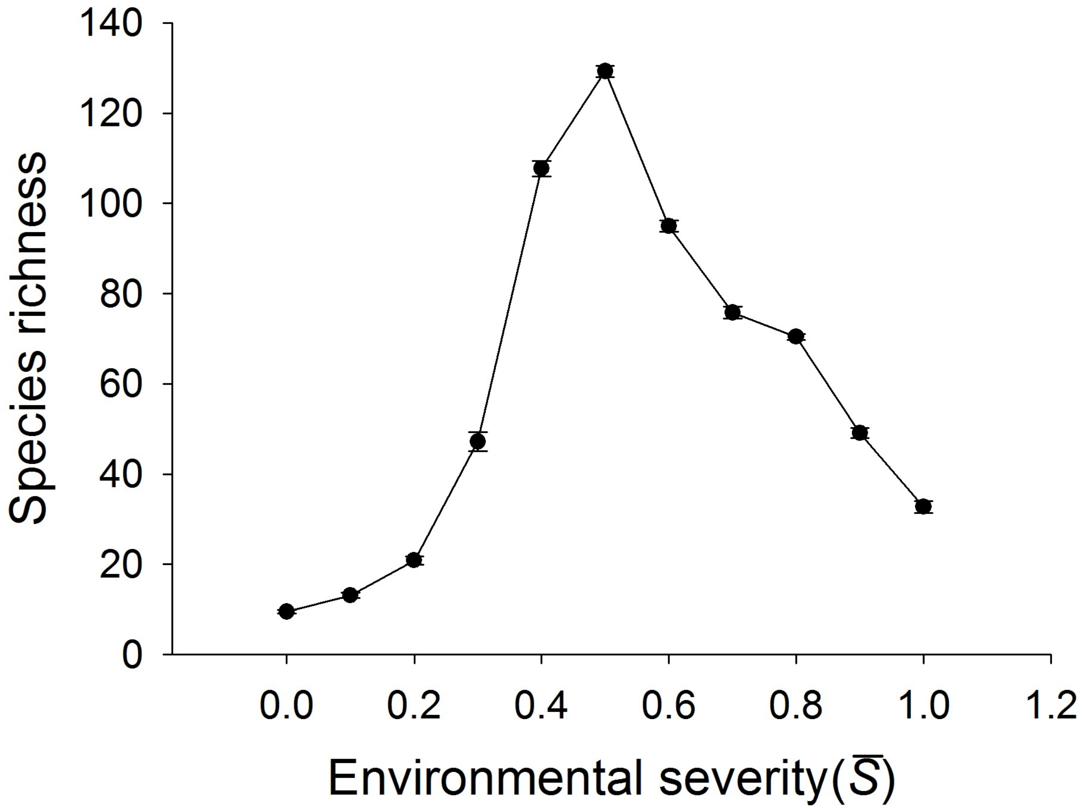

Supplementary Figure S3. Change of species richness along the environmental severity gradient. Landscapes were homogeneous with the size of 100 × 100 cells. Each data point in the panel represents the mean ± SE (N = 10). The parameter values used in models are r_{max} = 1, r_{min} = 0.2, r_s = 0.1 and c = 1.

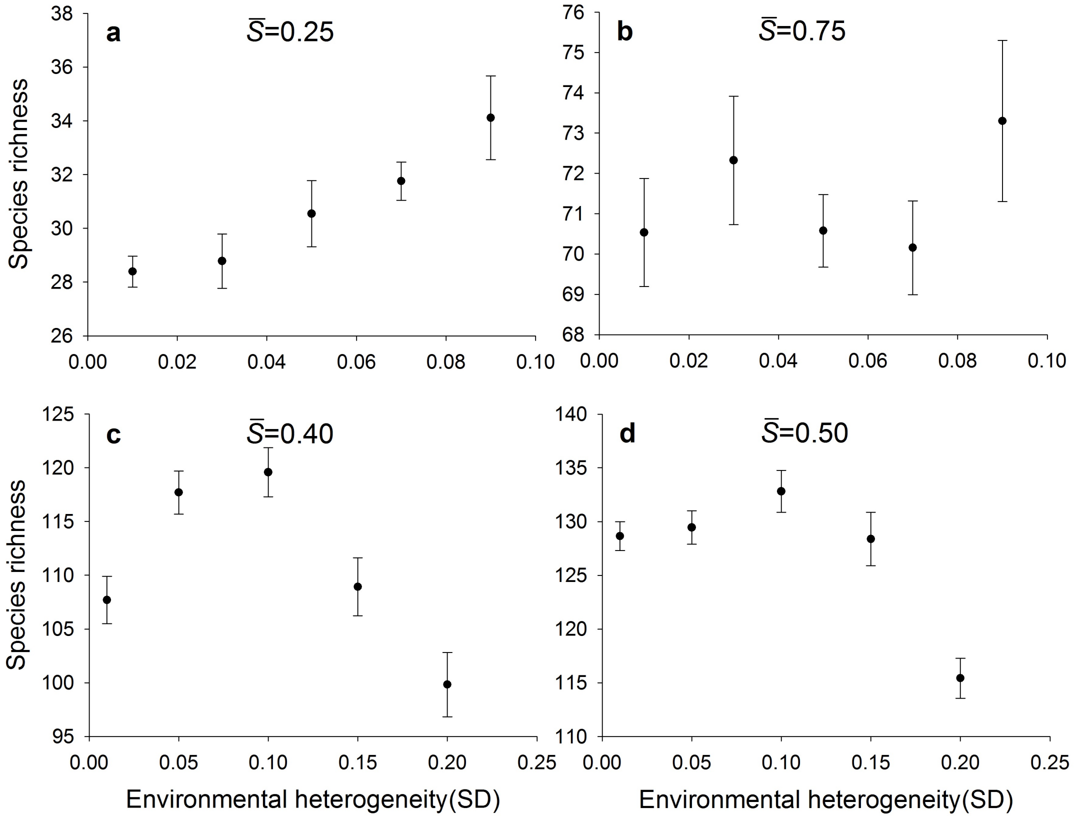

Supplementary Figure S4. Comparison of species richness-environmental heterogeneity relationships. Environmental heterogeneity was represented by the standard deviation of S_k values across patches. The environment severity ($\bar{S}$) was 0.25 in (**a**), 0.75 in (**b**), 0.40 in (**c**), and 0.50 in (**d**). The whole landscape was divided into 400 patch types. Each data point in panels represents the mean ± SE (N = 10).
